# Health economic analyses of latent tuberculosis infection screening and preventive treatment among people living with HIV in lower tuberculosis incidence settings: a systematic review

**DOI:** 10.12688/wellcomeopenres.16604.2

**Published:** 2023-01-05

**Authors:** Rebecca F. Baggaley, Carolin Vegvari, Christian A. Dimala, Marc Lipman, Robert F. Miller, James Brown, Svetlana Degtyareva, Helena A. White, T. Déirdre Hollingsworth, Manish Pareek

**Affiliations:** 1 Department of Population Health Sciences, University of Leicester, Leicester, LE1 7RH, UK; 2 Department of Infectious Disease Epidemiology, Imperial College London, London, UK; 3 UCL Respiratory, University College London, London, UK; 4 Royal Free London National Health Service Foundation Trust, London, UK; 5 RUDN University, Moscow, Russian Federation; 6 Department of Infection and HIV Medicine, University Hospitals of Leicester NHS Trust, Leicester, UK; 7 Big Data Institute, University of Oxford, Oxford, UK; 8 Oriole Global Health Ltd, London, UK; 9 Department of Respiratory Sciences, University of Leicester, Leicester, LE1 7RH, UK

**Keywords:** HIV; latent tuberculosis; screening; health economic; cost-effectiveness; cost-utility; model; review

## Abstract

**Introduction: **In lower tuberculosis (TB) incidence countries (<100 cases/100,000/year), screening and preventive treatment (PT) for latent TB infection (LTBI) among people living with HIV (PLWH) is often recommended, yet guidelines advising which groups to prioritise for screening can be contradictory and implementation patchy. Evidence of LTBI screening cost-effectiveness may improve uptake and health outcomes at reasonable cost.

**Methods: **Our systematic review assessed cost-effectiveness estimates of LTBI screening/PT strategies among PLWH in lower TB incidence countries to identify model-driving inputs and methodological differences. Databases were searched 1980-2020. Studies including health economic evaluation of LTBI screening of PLWH in lower TB incidence countries (<100 cases/100,000/year) were included.

**Results: **Of 2,644 articles screened, nine studies were included. Cost-effectiveness estimates of LTBI screening/PT for PLWH varied widely, with universal screening/PT found highly cost-effective by some studies, while only targeting to high-risk groups (such as those from mid/high TB incidence countries) deemed cost-effective by others. Cost-effectiveness of strategies screening all PLWH from studies published in the past five years varied from US$2828 to US$144,929/quality-adjusted life-year gained (2018 prices). Study quality varied, with inconsistent reporting of methods and results limiting comparability of studies. Cost-effectiveness varied markedly by screening guideline, with British HIV Association guidelines more cost-effective than NICE guidelines in the UK.

**Discussion: **Cost-effectiveness studies of LTBI screening/PT for PLWH in lower TB incidence settings are scarce, with large variations in methods and assumptions used, target populations and screening/PT strategies evaluated. The limited evidence suggests LTBI screening/PT may be cost-effective for some PLWH groups but further research is required, particularly on strategies targeting screening/PT to PLWH at higher risk. Standardisation of model descriptions and results reporting could facilitate reliable comparisons between studies, particularly to identify those factors driving the wide disparity between cost-effectiveness estimates.

**Registration:** PROSPERO CRD42020166338 (18/03/2020).

## Introduction

Nearly a quarter of the world’s population has latent TB infection (LTBI), meaning they are infected but do not (yet) have symptoms of tuberculosis (TB) and cannot transmit infection. Without antibiotics, approximately 5% of immunocompetent individuals acquiring LTBI progress to TB disease within the first two years following infection, and another 5% over the remainder of their lifetimes ^1, 2^. This risk is higher for people living with HIV (PLWH) and may remain elevated even with antiretroviral therapy (ART). While a 2010 systematic review estimated that approximately 30% of co-infected people may eventually develop TB disease, and these subjects were at increased risk of premature death ^3^, a UK study found incidence of TB disease during long-term ART to be much closer to background rates ^4^. It is therefore important to evaluate the costs and benefits of testing and treatment of LTBI for PLWH, yet little research has been published on the cost-effectiveness of LTBI screening and preventive treatment (PT, also referred to as chemoprophylaxis) for this group.

Earlier detection and PT of LTBI when patients are diagnosed with HIV or when they are receiving HIV care prevents progression to active disease, thereby reducing cost of TB care, TB-related morbidity and may also reduce onward TB infection transmission and costs of contact tracing. As well as these benefits to the patient and to the health system, treatment of patients with LTBI is also an important intervention for TB elimination, particularly for low-incidence countries where the long-lasting benefit of PT will not be mitigated by repeated TB re-exposure within the general population ^5– 9^. However, there is currently no consensus concerning which individuals to target for LTBI screening/PT: guidelines vary by low TB incidence country.

Many European countries test all HIV clinic attendees, either with the tuberculin skin test (TST) or interferon-gamma release assays (IGRA) ^10, 11^, while other countries favour a targeted approach. As TB incidence falls in low TB incidence settings, the contribution to active TB of those with reactivation of chronic latent infection increases, but the cost-effectiveness of LTBI screening/treatment falls. Targeting groups at higher risk of infection, for example migrants from endemic regions, may be more feasible and will maximise patient benefit while minimising government spending. For example, the British HIV Association (BHIVA) guidance advises testing with IGRA alone to all PLWH from high/medium TB incidence countries, and only screening those from low TB incidence countries (<40 TB cases/100,000 population) if additional risk factors for TB are present (listed in the guidance) ^12^. By contrast, the UK National Institute for Health and Care Excellence (NICE) recommends that all PLWH should be targeted for screening ^13^. Given this divergence in guidelines, compliance is reported to be low ^14^. A uniform, evidence-based national guideline for the UK is required ^15^.

We conducted a systematic review to evaluate whether health economic studies are comparable in their conclusions regarding the cost-effectiveness of LTBI screening/treatment for PLWH or targeting subpopulations of PLWH at higher risk of infection to improve this cost-effectiveness. We focussed on lower TB incidence countries only (<100 TB cases/100,000 population), as this incorporated both low incidence (<40/100,000) countries, which tend to be high-income, plus middle-income countries including Brazil and China, which share more in common with low TB incidence settings in terms of TB control than with high TB incidence settings. We aimed to assess which aspects of these economic evaluations, in terms of both model structure and model inputs, most influence their predictions and where knowledge gaps remain, in order to guide future research to provide the necessary evidence on which to base national guidelines.

## Methods

This study was registered on the International Prospective Register of Systematic Reviews ( PROSPERO) registration number CRD42020166338 (18/03/2020). It was conducted in accordance with PRISMA guidelines ^16^ (see *Reporting guidelines* ^17^).

### Selection criteria

To be eligible for inclusion, studies had to:

1) Include an intervention involving screening for LTBI among PLWH aware of their HIV status, and subsequent LTBI diagnosis and treatment. PLWH may or may not be receiving antiretroviral therapy (ART).
2) Include scenarios for a lower TB incidence country (<100 cases/100,000/year).
3) Report results of a health economic evaluation employing a modelling component. This could include decision tree, Markov, individual-based models or any other type of health economic model structure. Analyses required a health component (e.g., quality-adjusted life-years (QALYs) gained/disability-adjusted life-years (DALYs) lost, deaths averted) and a cost component.

Studies were excluded where:

1) The study population was not exclusively PLWH.
2) The intervention involved mass LTBI chemoprophylaxis of all PLWH rather than treatment only following a positive LTBI screening test.
3) The intervention involved screening of TB disease rather than latent TB infection.

Articles for inclusion had to be literature (peer-reviewed full papers or research letters in peer-reviewed journals). Abstracts, presentations, posters, non-research letters and editorials were excluded (these formats provide insufficient details on methods used). Reviews and grey literature were also excluded. No restrictions were placed on the modelled study population in terms of factors such as age, gender, ethnicity, health or treatment status. There was no study exclusion based on choice of comparison groups, but their suitability was assessed as part of the evaluation of study quality. There were no restrictions by date or language of publication.

### Search strategy and data extraction

We searched for published studies reporting the cost-effectiveness, cost-utility or cost-benefit of screening for LTBI among PLWH in lower TB incidence countries (defined as <100 cases per 100,000 population/year, WHO 2018 estimates ^18^). Ovid Embase, PubMed and Web of Science were searched for articles published between 1 ^st^ January 1980 and 30 ^th^ September 2020 (date of the most recent search) using terms for cost-effectiveness studies, tuberculosis, screening and HIV (see *Extended data* ^17^ for full search terms).

Two reviewers (RFB, CV) independently screened the papers at all levels: title, abstract and full-text. Discrepancies were discussed between the reviewers to reach a consensus, and where necessary, in consultation with co-authors. Bibliographies of articles passing the full-text screening were subsequently reviewed for any additional, relevant papers. A data extraction schedule was developed and used to retrieve information from included studies regarding aspects including: study characteristics (authors, publication year, conflicts of interest and funding statements), setting, characteristics of modelled population, interventions and comparators analysed, year/duration of study, data used for model inputs, model type (e.g., Markov, discrete event simulation), diagnosis methods (including sensitivity and specificity assumptions), latent and active TB positivity rates, LTBI reactivation rate, treatment uptake and completion rates, treatment effectiveness, health economic aspects including model time horizon, perspective adopted (e.g., health service, societal), health and cost discount rates applied, costs included (e.g., costs of screening, costs of treatment), health utilities, and the key results and conclusions of the study (e.g., total incremental costs, QALY/DALYs and incremental cost-effectiveness ratio (ICER) for each screening intervention). We extracted base case cost-effectiveness estimates plus other types of model outcome, and uncertainty bounds and sensitivity analysis methods. Data were extracted independently by two reviewers (RFB, CAD). For the purposes of this analysis, we did not contact authors for clarification because we aimed to evaluate the information that would be available to the reader, particularly policy and decision makers. All data were managed using a Microsoft Excel spreadsheet, and validated by an independent reviewer.

### Data analysis

Included studies were summarised according to study design, comparators and overall results. Studies were compared and assessed on the basis of study quality, perspective, design and parameter selection and valuation. Study reporting completeness was assessed using the Consolidated Health Economic Evaluation Reporting Standards (CHEERS) checklist ^19^ and study quality was assessed using the Gates Reference Case for Economic Evaluation ^20^ (RFB, CV). To aid comparability, costs were inflation-adjusted to 2018 in the local currency and then converted to US$ using consumer price indices and average annual exchange rates, using MS Excel version 2012 ^21– 23^. Forest plots were constructed using R version 4.0.3 to present study ICER (cost/QALY gained or cost/DALY averted) estimates. Cost-effectiveness studies may be more impactful by generating lower ICER values, so structural model assumptions which may particularly affect outputs, and therefore introduce bias, were evaluated. There were too few studies and lack of comparability between studies to employ further analysis by subgroup.

## Results

### Search results

**Figure 1.**
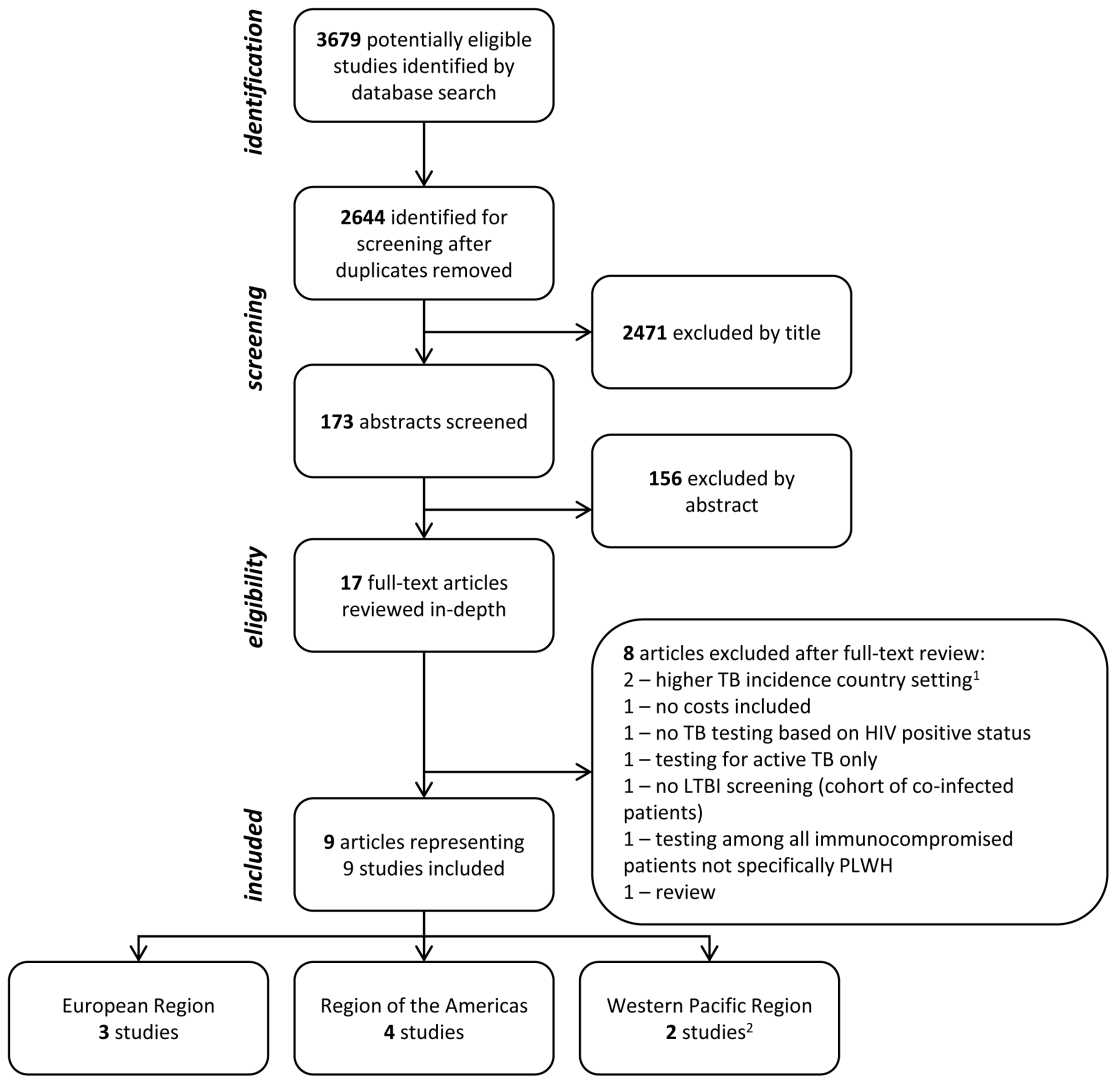
Flow chart of study selection, showing included studies stratified by World Health Organization region. The search identified 1627, 1261, and 791 potentially relevant titles from PubMed, Embase, Web of Science, respectively (3679 in total). Eight articles provided model-based cost-effectiveness estimates for the screening for and treatment of latent tuberculosis infection among people living with HIV in lower TB incidence countries and were included in the review. The search was conducted for articles published 1st January 1980 up to the 30 ^th^ September 2020. PLWH – people living with HIV; TB – tuberculosis. ^1^ Higher TB incidence countries defined as ≥150/100,000 cases as of 2018 ^18^). ^2^ Kowada *et al.* modelled “low TB incidence countries” (defined as <24 cases/100,000) but the majority of the input data are from Japan ^26^.

Database searches identified 2644 titles to screen after removing duplicates, resulting in 17 articles that went to full-text review ( Figure 1). Full-text review identified nine studies for inclusion and in-depth analysis ^24– 32^.

### Study characteristics

The main characteristics of included studies and the resulting ICERs are presented in Table 1 and epidemiological factors are summarised in Table 2. All studies were performed using dynamic-type Markov models with decision tree components except studies by Wong *et al.* (system dynamics model, similar to Markov) ^30^ and Jo *et al.* (individual-based transmission model) ^31^. The target populations were in the US ^27, 29, 31^, Italy ^28^, Japan ^26^, Brazil ^24^, UK ^25, 32^ and China (Hong Kong) ^30^. All studies included adult PLWH populations only, except Jo *et al.*, where age-associated inclusion criteria were not recorded ^31^. Time horizons of lifetime ^25, 27, 29, 32^, 30 years ^26, 31^, 20 years ^24^, and 10 years ^28^ were employed. All studies adopted a health service perspective except the study by Wong *et al.*, which did not report the time horizon or perspective ^30^.

**Table 1.** Summary of study characteristics and main findings of included economic evaluations of latent tuberculosis infection screening among people living with HIV in lower TB incidence countries. (Costs shown are in currencies and cost years as stated in the original publications.)

Study (author, year)	Setting	Population screened	Intervention/ alternatives	Model type	Perspective Model horizon Discount rate ^a^	Estimate year	WTP threshold assumed	Principal results	Conclusions
Sawert *et al.* 1998 ^28^	Italy TB: 7 ^b^ MDR: low ^b^ HIV: 0.2% ^b^	Hypothetical cohort of PLWH; 3 groups: 1) TST+, 2) anergic with various levels of immune suppression, 3) all PLWH	PT policy options: treating only: 1) TST+, 2a) TST+ and anergic with CD4 <200 cells/mm ^3^, 2b) TST+ and anergic with CD4 <350 cells/mm ^3^, 2c) TST+ and all anergic ^c^	Markov	Health service 10 years 3% per year	1997	NS ^d^	1) TST+: cost-saving (-US$7·7 million; 1153 [IQR 1026-1245] QALYs gained). Policies 2a-c increase life expectancy by extending PT to anergic patients and generally also lead to cost reduction.	PT for TST+ PLWH increases life expectancy and decreases medical costs. Its extension to anergic patients may be justifiable on economic grounds in populations with high TB prevalence
Linas *et al.* 2011 ^27^	USA TB: 2 ^b^ MDR: low ^b^ HIV: 0.4% ^b^	PLWH	TST vs IGRA vs no screening	Markov	Health service Lifetime 3% per year	2011	US$50,000 and US$100,000/QALYG	TST vs no testing: US$12,800/QALYG IGRA vs TST testing: US$23,800/QALYG	Screening should be prioritised for PLWH. IGRA is more cost-effective than TST screening
Kowada 2014 ^26^	Low TB incidence countries ^e^ TB: <24 ^e^ MDR: 0.012 ^e^ HIV: 0.4% ^b^	HIV-infected 20-year-old pregnant women	Different test scenarios: 1) TST, 2) QFT (IGRA), 3) T-SPOT (IGRA), 4) TST followed by QFT, 5) TST followed by T-SPOT Different targeting scenarios: 1) close contacts, 2) migrants from high TB burden countries, 3) occasional screenings ^f^	Markov	Health service 30 years 3% annual	2012	US$50,000/ QALYG	Base case (screening of close contacts): T-SPOT test was most cost-effective. TST followed by QFT were most cost-effective for screening of migrants and occasional screenings. (ICER values not stated)	IGRA recommended for TB screening of high-risk ^g^ pregnant PLWH in low TB incidence countries
Azadi *et al.* 2014 ^24^	Brazil TB: 45 ^b^ MDR: low ^b^ HIV: 0.6% ^b^	PLWH in Rio de Janeiro, at low risk of reinfection	PT of all TST+ PLWH with no signs of active TB vs usual care	Markov	Health service 1 year (interventions) 20 years (disease outcomes) 3% per year	2010	2010 Brazilian GDP per capita (US$11,700)	Intervention averts 1·14 discounted DALYs (1·57 undiscounted DALYs) per 100 people tested. Median ICER: US$2273 (IQR 1779-3135) per DALY averted (less than a quarter of Brazil’s 2010 GDP)	Intervention is highly cost-effective in the context of Brazil
Capocci *et al.* 2015 ^25^	UK TB: 7 ^b^ MDR: low ^b^ HIV: 0.2% ^b^	PLWH in London, ~1/3 originating from sub Saharan Africa	Screening based on 1) NICE and 2) BHIVA guidelines, 3) all PLWH, 4) no testing	Unclear	Health service Lifetime 3·5% per year	2011/12	€24,000/ QALYG	2000-2005 ICER values: BHIVA vs no testing: €6270/QALYG (95%UI 3482-7864) NICE vs BHIVA testing: €6998/QALYG (95%UI 4130-18,813) All attendees vs NICE testing: €33,473/QALYG (95%UI 6895-164,519) 2005-2010 ICER values: BHIVA: €9332/QALYG (95%UI 5396-11,958) NICE: €32,564/QALYG (95%UI 25,066-66,251) All attendees: €74,067/QALYG (95%UI 19,625-665,734)	BHIVA and NICE testing regimens missed cases but are cost-effective compared with no testing. Testing was more expensive over time, suggesting that alternative or more targeted TB testing strategies must be considered
Tasillo *et al.* 2017 ^29^	USA TB: 2 ^b^ MDR: low ^b^ HIV: 0.6% ^b^	Non US-born PLWH US residents	1) TST only, 2) IGRA only, 3) “confirm positive” (initial TST, IGRA confirmation for TST+), 4) “confirm negative” (initial IGRA, TST for all IGRA–, + on either test indicates LTBI)	Markov	Health service Lifetime 3% per year	2015	US$100,000/QALYG	TST only: Dominated IGRA only: US$35,000/QALYG Confirm positive: US$18,000/QALYG Confirm negative: US$63,000/QALYG	Testing for and treating LTBI among non-US born US PLWH is likely cost-effective
Wong *et al.* 2019 ^30^	China TB: 59 _b_ MDR: high _b_ HIV: <0.1% ^b^	PLWH in Hong Kong	Frequency of testing: 1) zero, 2) testing by risk factors, 3) biennial testing (all PLWH), 4) up to 3 tests (all PLWH), 5) annual testing (all PLWH) – current policy	System dynamics model ^h^	NR NR 3·5% per year	2017-2023	$50,000/ QALYG	For PLWH testing LTBI negative at baseline, no subsequent testing strategies were cost-effective under the assumed threshold. Most cost-effective testing strategy was annual LTBI testing by risk: $97,231/QALYG	Changing the current testing strategy to less intense testing strategies is likely to be cost-effective in the presence of an increased coverage of LTBI testing and treatment at baseline
Jo *et al.* 2021 ^31^	USA TB: 2 ^b^ MDR: low ^b^ HIV: 0.6% ^b^	PLWH in four states (California, Florida, New York, Texas)	Rapid screening/PT scale-up of PLWH and other risk groups ^i^ compared to baseline uptake rates	Individual-based TB infection transmission model	Health service 30 years 3% per year	2018	NS	$6695/QALYG – California $2828/QALYG – Florida $11265/QALYG – New York $4811/QALYG – Texas $9323/TB case averted – California $4428/TB case averted – Florida $15347/TB case averted – New York $6879/ TB case averted – Texas	Cost-effectiveness of screening/PT was highest for the PLWH risk group ^i^ in all states
Capocci *et al.* 2020 ^32^	UK TB: 7 ^b^ MDR: low ^b^ HIV: 0.2% ^b^	PLWH attending an ambulatory HIV clinic in London, UK	30 testing strategies (including active TB only) based on test (IGRA, TST, CXR, induced sputum), BHIVA/NICE guidelines, targeting to subpopulations (Black Africans, middle TB incidence countries)	Markov	Health service Lifetime 3.5% per year	2018/19	£20,000-£30,000 (NICE threshold)	Of 18 strategies reported in main publication: Screening all PLWH (various tests): $56,479-144,929/QALYG Targeted screening of PLWH from sub Saharan Africa or middle TB incidence countries: $23,098-47,540/QALYG BHIVA/NICE guidelines: $49,990-254,194/QALYG	Only strategies testing PLWH from sub Saharan Africa, or testing those from countries with TB incidence >40/100,000 with TST alone, were cost-effective

ART – antiretroviral therapy; BHIVA – British HIV Association; CXR – chest X-ray; GDP – Gross Domestic Product; ICER – incremental cost-effectiveness ratio; IGRA – interferon gamma release assay; INH – isoniazid; IQR – interquartile range; LTBI – latent tuberculosis infection; mo – months; Markov – Markov cohort simulation; NICE – National Institute for Health and Care Excellence; NS – not reported; PLWH – people living with HIV; PT – preventive therapy; QALY – quality-adjusted life year; QALYG – QALY gained; QFT – QuantiFERON-TB Gold In-Tube (IGRA test); MDR – multidrug resistant TB; T-SPOT – T-SPOT.TB (IGRA test); TST – tuberculin skin test; vs – versus; WHO – World Health Organization; WTP – willingness to pay; Z – pyrazinamide; + positive test result; – negative test result; 95%UI – 95% uncertainty interval. ^a^ In all included studies the discount rate applied to both costs and benefits.

^b^ Population-level estimates of infection burden, unless otherwise reported. TB incidence data: incidence of TB per 100,000 people per year, most recent year (2020); data from World Health Organization ^34^. Multidrug-resistant TB categorisation according to WHO identification of 30 “high burden” MDR TB countries ^35^, which includes China. HIV prevalence data: % of population ages 15–49 years, most recent year (2020 = Italy, Brazil; 2019 = United States); data from UNAIDS ^36^. UNAIDS HIV data were not available for China and the United Kingdom. HIV prevalence estimate for the UK represents all people living with HIV (all ages, estimate 106,890 ^37^) divided by population size for 2020 ^38^. HIV prevalence estimate for China represents an estimate of all people living with HIV taken from a recent review by Xu *et al.* ^39^, citing an official health report (all ages, estimate 1.045 million) divided by population size for 2020 ^40^.

^c^ Policy 3 involved universal PT for PLWH i.e. no LTBI screening element and so was excluded from the review (Sawert *et al.* reported that policy 3 increased costs and may even decrease mean life expectancy ^28^.)

^d^ No ICER threshold is stated but authors highlight in the sensitivity analysis scenarios which produce ICER values <$10,000/QALY gained.

^e^ Majority of input data from Japan. “Low incidence countries” defined as <24 cases per 100,000 people per year “as reported by the reports of the World Health Organization ^41^” although in this report low incidence is defined as countries with an incidence rate of <20 cases per 100,000 people per year or <10 cases in total. MDR rate for modelled scenarios reported as 0.012 (range 0-0.1) which represents the proportion of HIV positive pregnant women who had MDR-TB.

^f^ Frequency/schedule of “occasional screenings” scenario not defined.

^g^ ”High-risk” is not defined. Kowada reports that the US Centers for Disease Control and Prevention (CDC) states that high-risk women are “those with known or suspected TB contacts, injection drug use, HIV or other immunosuppression, foreign birth, and/or residence in congregate settings in low TB burden countries ^42^ “which implies that all pregnant PLWH are high-risk.

^h^ System dynamics models are similar to Markov models in being cohort-based but they allow interaction between different model entities e.g., infectious disease transmission models, where interactions between infected and uninfected individuals is important.

^i^ Other risk groups evaluated: non-US-born, diabetics, homeless, and incarcerated ^31^.

**Table 2.** Summary of key assumptions and study parameter values of included studies.

Study (author, year)	Data sources	ART and MDR assumptions	LTBI prevalence ^a^	Secondary TB infection transmission	Annual reactivation rate /active TB mortality	Test used (sensitivity, specificity)	Screening and PT uptake, adherence, completion	LTBI PT regimen and effectiveness ^b^	Adverse events	Utilities
Sawert *et al.* 1998 ^28^	Prospective cohort study, including cost data	Pre-ART setting 3-5% MDR – tx costs 10-fold higher	6.6-21.1% ^c^	10 new infections per untreated active TB case; 2 new infections per treated case	CD4 >350: 2%/year CD4 200-350: 8%/year CD4 <200: 12%/ year Mortality: CD4 ≥200: 25%/ year CD4 <200: 36%/ year	TST (NR, NR)	Screening uptake: NR 75% PT adherence ^d^	INH 12mo No DILI: 85-95% Post DILI: 25% ^e^	DILI: 0.3-6.4%	Not reported ^f^
Linas *et al.* 2011 ^27^	Published literature including CDC surveillance data and National Health and Nutrition Examination Survey for TST positivity rate	NR MDR not included	5.3% (range 2-9%)	Each case of reactivation TB resulted in 0.31 (0.25- 1.1) cases of secondary TB distributed throughout the expected lifetime of contact cases	2.07%/year Mortality: 5% risk (no comorbidities), 6% risk (other chronic conditions), over 6 months	TST (89% [50-100%], 98% US- and 92% non- US-born ^g^ [50-100%]) IGRA (83% [50-100%], 99% [50-100%])	Screening uptake NR; 88% returned to receive TST result (PLWH not returning were ineligible for PT). 90% PT uptake for those returning with a positive TST test (uptake for IGRA test group NR) 52% completion	INH 9mo Full course: 90% (75- 100%) 6-8mo: 60% (50-75%) 3-5mo: 30% (0-69%)	DILI (<34y): 0.1% (0.05-0.15%) DILI (≥35y): 1% (0.5-1.5%) DILI mortality: 1% (0.5-1.5%)	LTBI state: 1 INH tx without toxicity: 1 (0.9- 1.0) Active TB state: 0.80 (0.6-1.0) Non-fatal DILI: 0.85 (0.6-1.0) (1 month) Month of TB or DILI death: 0.3 (0.2-0.5) After having active TB: 1.0 (0.9-1.0)
Kowada 2014 ^26^	Published literature	NR 1.2% (0-10%) MDR – higher mortality and morbidity rates, >10-fold higher tx costs	7-36% during pregnancy 11-55% postpartum	Not included	0.02-1.8%/year during pregnancy 0.03-2.7%/year during postpartum Mortality: All-cause mortality: 0.00091 (20 years), 0.0013 (30 years), 0.0026 (40 years) Increased mortality due to active TB: 5.2 (95%CI 1.7- 15.6) Mortality rate, MDR TB: 0.13 (95%CI 0.06-0.26)	TST (43%, 97% (non- BCG), 59% (BCG)) IGRA (61% QFT 65% T-SPOT, 99% QFT 98% T-SPOT)	Screening uptake: NR 80% PT adherence (IGRA) 50% PT adherence (TST) ^h, i^	INH 6mo 68%	DILI: 1.1%	Non-LTBI, non- TB: 1 ^j^ LTBI, no tx: 1 LTBI, tx, no adverse events: 0.99 LTBI, tx, DILI: 0.85 Active TB, non- MDR (pre and during tx): 0.80 (no range) Active TB, MDR (pre and during tx): 0.58 (no range)
Azadi *et al.* 2014 ^24^	Cluster- randomised trial 2005-2009 providing TST and IPT to PLWH in 29 HIV clinics in Rio de Janeiro (THRio study)	Majority (67%) of cohort on ART when initiating PT. Of the remainder, 35% initiated ART at some point during PT. MDR not included.	NR	Not included	4.8%/year ^k^ Mortality: 1.3 TB deaths over 20 years among 100 PLWH patients ^l^	TST (NR, NR)	NR	INH 6mo 87% effectiveness ^m^	Not included	TB/HIV co- infected: 72.78 TB-infected: 74.09 HIV-infected: 77.40 ^n^
Capocci *et al.* 2015 ^25^	10 year follow-up data (2000- 2010) from a large London, UK HIV clinical cohort	Model parameterised for 2000-2005 and 2005- 2010 to reflect change in ART coverage in UK over time. ART coverage: 61% (2000), 74% (2005), 86% (2010). ^o^ Effect of tx resistance implicitly explored in sensitivity analysis by varying PT effectiveness and tx costs.	Black Africans: 13% Middle TB incidence countries: 10% Low TB incidence countries: 3%	Not included	NR Mortality: NR	TST (NR, NR) IGRA (91% [70-100%], NR)	Screening uptake: 87% (87-100%) 87% (60-100%) PT uptake (remainder stated to have declined or failed to complete PT) ^p^	INH 6mo 62% (40-100%)	Not included	Quality of life decrements: Active TB: 0.676 (0.271-6.72) ^q^ LTBI: 0.007 (0.001-0.1)
Tasillo *et al.* 2017 ^29^	Published literature	NR MDR not included.	15.9% (range 0-100%)	Secondary infections (first generation only). 0.250 cases (0.1-1.0) (units not specified)	10% (range 5-20%) lifetime risk Mortality: 0.05 (range 0.025- 0.075) ^r^	TST (67% [50-100%], 87% [50-100%]) IGRA (77% [50-100%], 99% [50-100%])	Screening uptake: NR; return for TST result: 82% (0-100%) PT uptake: 90% (50-100%) PT completion: 78.3% (50- 100%)	INH + rifapentine 3mo 90% (50-100%)	DILI: 0.5% (0.0- 1.0%) (DILI mortality: 0.1% (0.0-0.2%))	LTBI: 1 (0.99-1.0) DILI: 0.750 (0.6- 1.0) Active TB: 0.83 (0.75-1.0) Post-TB: 1 (0.87- 1.0)
Wong *et al.* 2019 ^30^	15-year longitudinal clinic data. Patients diagnosed 2002-2017	ART coverage varied in scenarios between baseline (80%) and 100%. 100% ART coverage assumed following active TB diagnosis. 1.6% MDR – higher morbidity, nearly 10-fold higher tx costs	26.2% ^s^	Not included	Pre-ART: Non-locals, ^t^ CD4 <200: 39.1%/year Non-locals CD4 >=200: 20.9%/year Locals CD4 <200: 10.7%/year Locals CD4 >=200: 7.9%/year On ART: Non-locals, CD4 <200: 42.9%/year Non-locals CD4 >=200: 9.1%/year Locals CD4 <200: 9.5%/year Locals CD4 >=200: 2.3%/year Mortality: 0.0001 ^r^	TST (NR, NR)	LTBI screening uptake: 44-65% (first year) 39-66%/year (subsequent years) PT uptake: 44- 76% (varied by study year)	INH 9mo Pre-ART: TB reactivation reduced to 0–0.0051 cases/py On ART: TB reactivation reduced to 0–0.0196 cases/py (range depends on CD4 count and locals vs non-locals) ^u^	Not included	Without TB = 1 ^v^ Active TB, CD4 ≥200: 0.83 Active TB, CD4 <200: 0.702 MDR-TB: 0.68
Jo *et al.* 2021 ^31^	Published literature	Assumed PLWH population receiving ART MDR not included	Calibrated using national TB surveillance data stratified by state, ethnicity, age, and 5-year time periods	Transmission dynamic model. Average number of transmissions per active TB case calibrated to state-specific TB incidence (which decayed over time)	Calibrated using national TB surveillance data, assuming exponential decline in reactivation rate over time and higher rate with older age 9.2% active TB case fatality	IGRA (85%, NR)	Screening uptake: 100% PT uptake: 85% PT completion: 78%	INH + rifapentine 3mo 93%	3.2% without hospitalisation, 0.015% with hospitalisation	LTBI: 0.97 Active TB: 0.76 HIV state (assuming asymptomatic with ART): 0.94 PT toxicity (no hospitalisation): 0.75 ^w^ PT toxicity (hospitalisation): 0.5 QALY losses: Active (non-fatal) TB: 0.12 Mean loss due to PT toxicity: 0.002
Capocci *et al.* 2020 ^32^	HIV clinical cohort plus published literature	95% clinic population parameterising the model were on ART; BHIVA guidelines strategy based on duration of ART use MDR not included	9% tested subjects	0.2 secondary active TB cases prevented by averting each active case of TB (0.4, 1.0 and 2.0 explored in sensitivity analysis)	Lifetime risk of active TB: IGRA+: 10% TST+/IGRA–: 2% TST–/IGRA–: 0.02% ^x^ (92/100,000 lifetime reactivation risk for PLWH in England and Wales) Mortality NR	TST (NR, NR) IGRA (NR, NR)	TST return rate for those having TSTs as well as IGRAs: 53% (30-90%) PT uptake: 50% (35-65%) PT completion: NR	INH 6mo or INH + rifampicin 3mo 62% (59-65%)	Not included	Quality of life decrements: Active TB: 0.676 LTBI: 0.007 Tx asymptomatic active TB: 0.2

ART – antiretroviral therapy; BCG - Bacillus Calmette-Guérin; BHIVA – British HIV Association; CBA – cost-benefit analysis; CD4 – CD4 count (cells/mm ^3^); CE – cost-effectiveness; CEA – cost-effectiveness analysis; CUA – cost-utility analysis; DILI – drug-induced liver injury; EE – economic evaluation; GDP – Gross Domestic Product; HIV+ – HIV-infected; ICER – incremental cost-effectiveness ratio; IGRA – interferon gamma release assay; INH – isoniazid; LTBI – latent tuberculosis infection; MDR – multidrug resistant; mo – months; NR – not reported; OI – opportunistic infection; PT – preventive therapy for LTBI; py – person-year; TST – tuberculin skin test; tx – treatment; y – years; Z – Pyrazinamide.

^a^ Prevalence of LTBI among PLWH.

^b^ Unless otherwise stated, effectiveness is of completed regimen.

^c^ Calculated based on reported TB prevalence among tuberculin-positive, tuberculin-negative non-anergic and anergic patients.

^d^ Non-adherers are assumed to be experience zero PT effectiveness and zero frequency of adverse events.

^e^ Assumes DILI occurs on average during third month of preventive therapy.

^f^ Sawert *et al.* 1998 ^28^ state that they used "medians of recently published QoL adjustment factors for various levels of immunosuppression in HIV infection ^43^".

^g^ Lower specificity for non-US-born due to Bacillus Calmette-Guérin (BCG) vaccination.

^h^ No justification for large difference in adherence rates between TST-positive and IGRA-positive PLWH provided.

^i^ No information on how adherence relates to PT effectiveness and adverse events.

^j^ All states are among pregnant PLWH.

^k^ Assuming 11.5 TB cases over 20 years among 100 PLWH patients (with 12% LTBI prevalence).

^l^Majority of PLHIV are not LTBI-infected; mortality of individuals with active TB not stated.

^m^An additional effect of PT reducing TB mortality (by 17%) in addition to reducing TB incidence was explored in sensitivity analysis.

^n ^Assumed individuals experience the TB/HIV co-infected disability state for 1 year before reverting to the disability state of chronic HIV.

^o^ In addition, for the scenario using BHIVA guidelines, LTBI testing is dependent on duration of ART use. Recommended LTBI testing for PLWH from sub Saharan Africa if duration on ART <2 years; from a middle TB incidence country and CD4 count <500 cells/mm ^3^ and duration on ART <2 years; and from a low TB incidence country and CD4 count <350 cells/mm ^3^ and duration on ART <6 months ^25^.

^p^ Suboptimal adherence is accounted for through lower estimates of PT effectiveness.

^q^As stated in the publication.

^r ^No units stated.

^s^ Wong *et al.* report that 26.2% of those tested for LTBI were positive among their cohort but LTBI prevalence reported for local and non-local PLWH populations in Supplementary Online Content does not tally with this ^30^.

^t^ “Nonlocal” infections are defined as “infections in non-Chinese individuals and residents without right of abode”.

^u ^PT effectiveness: Pre-ART: Non-locals: reactivation reduced to 0, all CD4 counts. Locals: reduced to zero for CD4 >=200 cells/mm ^3^; reduced to 0.0051 cases/py for CD4 <200 cells/mm ^3^ (21-fold reduction). On ART: Non-locals: reduced to zero for CD4 >=200 cells/mm ^3^; reduced to 0.0196 cases/py for CD4 <200 cells/mm ^3^ (22-fold reduction); Locals CD4 <200 cells/mm ^3^: 0.0018 cases/py (53-fold reduction); Locals CD4 >=200 cells/mm ^3^: 0.0025 (9-fold reduction).

^v^Utility = 1 for TB-uninfected PLHIV regardless of CD4 count.

^w^Stated as 0.25 in Jo *et al.* ^31^ but from review of the source publication, this represents the utility decrement rather than the utility weight ^44^.

^x^Stated as 0.2% for patients testing TST– in the Supplementary Material ^32^.

***LTBI screening strategies.*** Four studies included screening comparisons between TST and IGRA tests ^26, 27, 29, 32^; and three evaluated testing schedules that involved both tests ^25, 29, 32^. The remainder evaluated TST only ^24, 28^. PT regimens modelled were six-month ^24– 26, 32^, nine-month ^27, 30^, and 12-month ^28^ isoniazid, and isoniazid plus rifapentine for three months ^29, 31^. Capocci *et al.* 2020’s analysis was informed by HIV clinical cohort data, where patients received six-month isoniazid or three-month isoniazid plus rifampicin depending on drug interactions ^32^. Studies investigated a number of different screening strategies for a wide range of PLWH target populations ( Table 1).

Counterfactuals were generally usual care (Azadi *et al.* used outcomes from public HIV care clinics not randomised to receive the LTBI screening/PT intervention ^24^; Wong *et al.*, annual LTBI diagnoses taken from clinical data ^30^; Jo *et al.*, baseline screening/PT levels previously estimated ^31, 33^) or zero testing ^25, 27– 29, 32^. Kowada *et al.* evaluated screening/PT for HIV-infected pregnant women only, but did not compare strategies targeting screening/PT to different populations (close contacts, migrants from high TB burden countries, “occasional screenings”), keeping each analysis independent and comparing only costs and benefits for each test type used ^26^. The author used the most cost-effective testing strategy as the base case for each scenario, so all other ICER values presented were dominated.

One study specified that LTBI screening was undertaken at HIV diagnosis and annually thereafter ^30^; other studies modelled screening of populations in established HIV care ^24, 25, 31^ or this was not recorded but is likely also to have been established care ^26– 29^. Capocci *et al.* 2020 stated that the population on which their model was based was offered LTBI screening at their next routine appointment for those in established care, as well as all newly HIV-diagnosed patients ^32^.

***Screening and treatment parameters.*** Two-thirds of studies did not report or incompletely reported test sensitivity and specificity values used ^24, 25, 28, 30– 32^ ( Table 2). For those studies reporting, TST sensitivity was 43–89% and specificity was 59–92%. IGRA sensitivity was 61%–83% while specificity was consistent at 98–99%. TST specificity is known to vary by BCG inoculation status, but only one study accounted for this (97% specificity for non-BCG-vaccinated individuals, 59% for vaccinated individuals ^26^). A further study stratified specificity by country of origin to reflect this difference implicitly (98% for US-born, 92% for non-US-born ^27^). The remaining study assumed 87% specificity ^29^.

Assumed effectiveness of full-course PT with isoniazid (INH) for six months was 62–68% ^25, 26^ (effectiveness assumptions were unclear in the study by Azadi *et al.* ^24^), while nine-month effectiveness was assumed to be 90% for one study ^27^, while a second study assumed differential effectiveness by CD4 count and region of origin (locals versus non-locals) ^30^. Effectiveness of twelve-month INH and three-month INH + rifapentine were estimated as 85–95% ^28^ and 90–93% ^29, 31^, respectively. Capocci *et al.* 2020 assumed 62% effectiveness for a cohort receiving either six-month INH or three-month INH + rifampicin, depending on drug interactions ^32^.

PT adherence was reported heterogeneously. Some studies reported adherence levels (Sawert *et al.* ^28^; Kowada *et al.* used different adherence levels depending on the test used ^26^) while others reported PT uptake coverage and proportion completing the PT course ^27, 29, 31^, but how adherence related to PT effectiveness varied and was not always clear. For example, Sawert *et al.* assumed non-adherers had zero PT effectiveness and zero adverse events ^28^. Linas *et al.* modelled PT effectiveness as a function of length of PT received (3–5, 6–8, full-course nine months) but did not state how their assumed 52% completion rate for PLWH translated into these lengths ^27^. Kowada *et al.* assumed strikingly different PT adherence for PLWH using the IGRA (80%) and TST (50%) tests, without explanation for this difference or how this affected PT effectiveness ^26^.

Adverse events were included by only five studies ( Table 2 ^26– 29, 31^) (drug-induced liver injury (DILI) only ^26– 29^, not specified by Jo *et al.* ^31^). Adverse event prevalence ranged from 0.1% (Linas *et al.* <34-year PLWH ^27^) to a range 0.3–6.4% ^28^. DILI-related mortality was accounted for in two studies (Linas *et al.* 1% ^27^, Tasillo *et al.* 0.1% ^29^) and a quality of life impact for four studies ^26, 27, 29, 31^ (utility values not reported by Sawert *et al.* ^28^).

***Epidemiological parameters.*** A wide range of LTBI prevalence estimates for the target populations were used, from 5.3% (PLWH in the US ^27^) to a range as high as 11–55% (postpartum women in low TB incidence countries ^26^) ( Table 2). Jo *et al.* calibrated both LTBI prevalence and reactivation rate of LTBI to TB disease using TB incidence data, with values not explicitly reported ^31^. Reported reactivation rates were also heterogeneous, with values of around 2%/year for PLWH with high CD4 counts in some studies ^26– 28^ and lifetime risk 10% ^29, 32^, to extremely large values of 8–21%/year even for PLWH at high CD4 counts ^30^.

Secondary TB transmission was included in five of the nine studies ^27– 29, 31, 32^, all including only first generation transmission but assuming different transmission rates, with the exception of Jo *et al.*, who employed a full TB infection transmission model ^31^. Again, the model parameter, average number of transmissions per active TB case, was calibrated to state-specific TB incidence levels (which decayed over time), but values for this decline were not reported. TB-related mortality also varied considerably, being far lower in the ART era than rates assumed by Sawert *et al.* in the absence of ART ^28^, although Jo *et al.* used a notably high 9.2% active TB case fatality ^31^. Incorporating secondary transmission will improve estimation of cost-effectiveness, as both the costs and health benefits of preventing secondary TB infections are taken into account. However, the magnitude of this impact depends on many model parameters including reactivation rate, infectiousness of people with active TB, and the treatment costs, morbidity and mortality of active TB infection. Recording of these epidemiological parameters was incomplete for some studies ^24, 25, 32^ ( Table 2). Therefore there is no clear relationship evident between incorporation of secondary TB transmission and cost-effectiveness estimation.

Three studies accounted for multi-drug resistance (MDR) ^26, 28, 30^, all of which assumed around 10-fold higher treatment costs for active TB and two of which assumed higher morbidity and/or mortality ^26, 30^. In addition, Capocci *et al.* 2015 stated that they implicitly incorporated the impact of treatment resistance into their treatment effectiveness estimate ^25^. Three studies did not explicitly incorporate ART ( Table 2). ART use would be expected to reduce cost-effectiveness estimates; it reduces health benefits of the intervention because TB progression rates and active TB-related mortality is vastly reduced for PLWH on ART ^45^. The one study parameterised based on the pre-ART era found LTBI screening/PT to be cost-saving ^28^. In addition, HIV treatment and care costs continue for life; therefore, for PLWH whose lives are saved by preventing TB-related mortality, these costs continue to accrue over their lifetime. However, of the four studies explicitly incorporating the health impact of ART ^24, 25, 29, 30^, only one included HIV care/ART costs in their analysis ^29^.

***Utility (quality of life) values.*** All studies used QALYs as the principal health outcome measure except Azadi *et al.*, who used DALYs for their study based in Brazil ^24^. One study did not report utility values ^28^. While some studies assumed LTBI had no impact on utility values for PLWH ^26, 27, 29^, others assumed a small decrement ^25, 31, 32^. TB disease was associated with a 0.17–0.20 ^26, 27, 30, 31^ utility decrement except for Azadi *et al.* (assumed a very small difference in disability weights between TB-HIV coinfected and HIV-infected individuals: 72.78 and 77.40, respectively ^24^) and Capocci *et al.* (0.676 decrement ^25, 32^). Kowada *et al.* assumed a utility of 1 for all PLWH uninfected with TB, even for PLWH with low CD4 counts ^26^. Other utility decrements included by some studies included adverse events ^26, 27, 29, 31^ and MDR ^26, 30^.

***Costs.*** Key cost components are shown in Table 3. Despite adjusting for cost year, ranges for full-course LTBI PT ($103–1333), adverse event management ($289–12,987), TB disease treatment ($741–18,565) and per screening test (TST $8.28–46.51, IGRA $57.60–104.76) were large. Tasillo *et al.* included monthly healthcare costs for HIV ($2061, range $1030–3091 ^29^) while three studies assumed 10-fold higher treatment costs for MDR TB) ^26, 28, 30^. Capocci *et al.* 2020 included costs associated with asymptomatic, smear negative, culture positive TB ($1816) ^32^ but full details of estimation were not reported. 

**Table 3.** Key cost components (average per screened/treated patient) ^a^ extracted from included studies (adjusted to 2018 USD ^b^). Where available, reported uncertainty bounds are also shown in brackets.

Study (author, year)	Full-course LTBI chemoprophylaxis	Adverse event management	Active TB treatment	TST/IGRA testing
Sawert *et al.* 1998 ^28^	$357 (NS)	$393 (NS) ^c^	$7169 (NS)	$10.22 (NS)/NA
Linas *et al.* 2011 ^27^	$514 ($260-$781)	$289 ($144-$434) ^c^	$15,920 ($7900-$23,923)	$46.51 ($22-$70)/$57.60 ($29-$116)
Kowada 2014 ^26^	$563 (NS)	$12,987 ^c^ (NS)	$18,565 ^d^ ($16,234-$35,660)	$16.80 (NS)/$66.12 (NS)
Azadi *et al.* 2014 ^24^	$103 (NS)	Not included	$741 (NS)	$31.92 (NS)/NA
Capocci *et al.* 2015 ^25^	$1333 ($635-$2232)	Unclear	$12,917 ($6459-$25,834)	$27.36 ($14-$55)/$104.76 ($40-$157)
Tasillo *et al.* 2017 ^29^	$612 ($315-$1052)	$354 ^c^ ($277-$540)	$16,693 ^e^ ($3812-$32,733)	$8.28 ($5-$16)/$88.71 ($53-$105)
Wong *et al.* 2019 ^30^	$322 (NS)	Not included	$12,245 ^d^ (NS)	$20.15 (NS)/NA
Jo *et al.* 2021 ^31^	$394-451 ^f^	$216-247 ^c, f^	$10,574-22,565 ^f, g^	NA/$75-85 ^f^
Capocci *et al.* 2020 ^32^	$969 ($485-$1939)	Not included	$14,082 ($7041-$28,164)	$26.94 ($13-$54)/$77.64 ($39-$155)

IGRA – interferon gamma release assay; NA – not applicable (test not included in the analysis); Not included – cost of test not included in the analysis; NS – uncertainty interval not stated; TST – tuberculin skin test; tx – treatment. ^a^ List is not exhaustive.

^b^ Prices uplifted to 2018 US prices (most recent data) using the US Bureau of Economic Analysis (BEA) Price Index for Personal Consumption Expenditures by Function – Health ^22^. Costs for Capocci *et al.* 2015 ^25^ were converted to Great British pounds using the exchange rate €1=£0.83 used in the publication, uplifted to 2018 UK prices using the UK Consumer Price Index of Health ^21^, then converted to USD using the OECD purchasing power parity rate in 2018 (£0.687=US$1 ^23^). Costs for Capocci *et al.* 2020 ^32^ were also converted to USD using the 2018 OECD purchasing power parity rate ^23^.

^c^ Adverse events included were drug-induced liver injury (DILI) only for Sawert *et al.*, ^28^ Linas *et al.*, ^27^ Kowada *et al.* ^26^ and Tasillo *et al*. ^29^ Sawert *et al.* assumed 10% of DILI patients required hospitalisation ^28^. Linas *et al.* assumed hospitalisation for fatal DILI cases (case fatality 1%) but unclear what proportion of non-fatal DILI cases required hospitalisation ^27^ – we have assumed 0%. Tasillo *et al.* assumed excess costs for fatal DILI cases (case fatality 0.1%) ^29^. Jo *et al.* assumed 0.5% of adverse events required hospitalisation ^31^.

^d^ Average of costs to treat multidrug resistant (MDR) and non-MDR active TB. Uncertainty range is based on percentage MDR varying between 0% and 10% of all infections.

^e^ Average of treatment for non-severe and severe (requiring hospitalisation) active TB.

^f^ Costs varied by US state.

^g^ Probability of hospitalisation with active TB assumed to be 49%.

**Figure 2.**
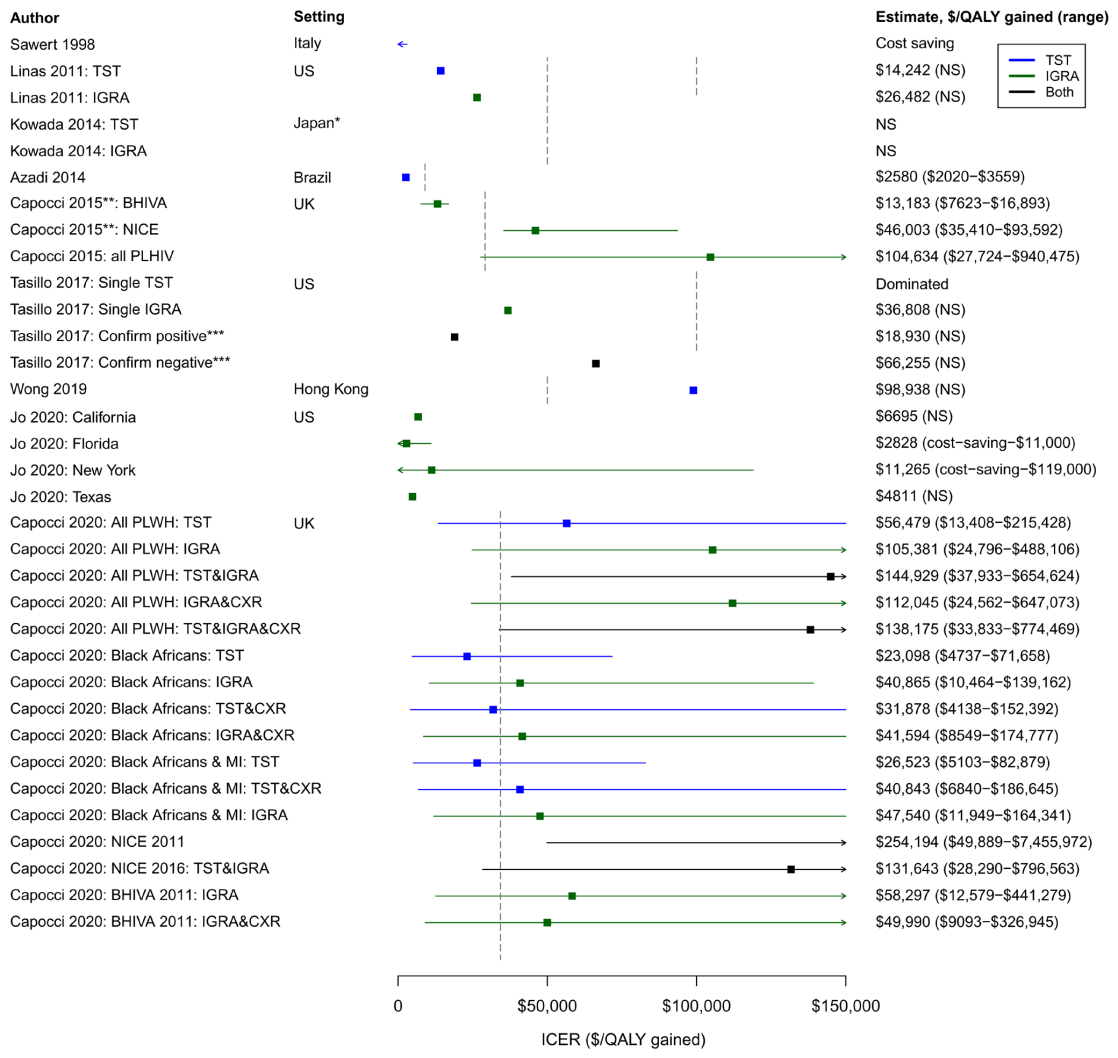
Forest plot of included study incremental cost-effectiveness ratio estimates (ICERs) adjusted to 2018 prices. Vertical dashed lines show willingness to pay (WTP) thresholds used in the included papers: US$100,000 ^27, 29^, US$50,000, ^26, 27, 30^, £30,000 ^32^ (converted to US$43,668), and €24,000 ^25^ (converted to US$29,112) (these thresholds have not been uplifted to 2018 values because thresholds tend to remain fixed rather increasing with inflation). Interventions with cost-effectiveness estimates below a WTP threshold are interpreted as being value for money. Azadi *et al.* used the 2010 Brazilian GDP per capita (US$11,700) as the WTP threshold ^24^. As this represents a more dynamic threshold, the value US$9001 is plotted, representing Brazil’s GDP per capita in 2018 ^46^. For Capocci *et al.* 2020, only the 16 strategies reported in the main publication are shown (excludes no testing scenario and chest X-ray only scenario) ^32^. CXR – chest X-ray; MI – middle TB incidence countries (40-300/100,000); NS – not stated; PLWH – people living with HIV; TST - tuberculin skin test; IGRA - interferon-gamma release assays. * “Low TB incidence countries” (majority of data taken from Japan). ** NICE recommendations: use both IGRA and TST if CD4 count <200 cells/mm ^3^, IGRA with or without concurrent TST if CD4 200-500 cells/mm ^3^. BHIVA recommendations are to use IGRA test. ICERs shown are 2005-2010 estimates (2000-2005 estimates not shown) ^25^. *** Confirm positive strategy: initial TST test, IGRA confirmation for TST positives. Confirm negative strategy: initial IGRA test, TST for all IGRA negatives, positive result on either test indicates LTBI.

***Main findings.*** The diversity of model assumptions and parameter values only partly explain the diverse results from these studies. Figure 2 summarises the ICER estimates each included study reported for various LTBI screening/PT strategies, alongside willingness to pay (WTP) estimates quoted or discussed by each study. In general, studies found that at least one screening/PT strategy evaluated was cost-effective according to their setting-specific threshold ( Figure 2 and Table 1) except Wong *et al.* because they evaluated strategies only for PLWH who tested TST-negative at baseline ^30^. Of the testing strategies evaluated, both Tasillo and Linas *et al.* concluded that strategies involving IGRA testing for PLWH were most cost-effective ^27, 29^.

Capocci *et al.* 2015 concluded that for the UK, only strategies targeting LTBI screening to higher risk PLWH (as defined by NICE and BHIVA guidelines) were cost-effective in 2000–2005, but these strategies became more expensive (likely due to increased ART coverage and/or proportionally fewer PLWH from high TB incidence countries), so by 2005–2010 only the BHIVA targeting strategy (higher-risk PLWH defined by country of origin, CD4 count and ART duration) was cost-effective ^25^. Their later paper included updated NICE guidelines and found that the most cost-effective strategies were not those based on UK guidelines, but involved targeting screening/PT to PLWH with country of origin in sub Saharan Africa and/or mid-high TB incidence countries ^32^. In contrast, Jo *et al.* reported extremely favourable cost-effectiveness estimates for screening/PT to all PLWH in four US states ^31^. Factors contributing to this large difference include the high TB disease case fatality assumed by Jo *et al.* (9.2%) and the high cost of LTBI PT assumed by Capocci *et al.* ( Table 3). Overall, the heterogeneity in model assumptions and parameter values we have described make further comparisons between study estimates difficult.

### Sensitivity analysis

All studies provided a univariate (one-way) sensitivity analysis using a selection of model parameters, and all but one ^27^ undertook probabilistic sensitivity analysis (PSA, where all or selected parameters are varied simultaneously within their parametric distribution to produce a range of plausible values for the ICER) ( Table 4). However, choice and number of parameters included in analyses varied and were selected subjectively. Systematic presentation of the most influential parameters on model outcomes were attempted by four studies (as a table ^25, 32^ or as a Tornado plot ^24, 31^, albeit with only three parameters for Azadi *et al.* ^24^). PSA was generally used to create cost-effectiveness acceptability curves (CEACs) only in earlier studies, showing the strategies by WTP threshold, but more recent studies employed a more systematic, comprehensive approach to SA including presentation of ICER estimates with uncertainty intervals ^31, 32^.

**Table 4.** Summary of sensitivity analyses employed in each included study (green cell = included in the analysis, red cell = not conducted/reported).

Study (author, year)	One-way	Tornado plot	Two-way	PSA	Uncertainty bounds for results
Sawert *et al.* 1998 ^28^	Varied compliance only.	X	Varied compliance and LTBI prevalence for anergic and tuberculin+ PLWH	LTBI prevalence by TST result and anergy status, PT effectiveness, proportion experiencing DILI, DILI mortality rate, life expectancy by TB status and CD4 count	✓
Linas *et al.* 2011 ^27^	Series of one-way and two-way sensitivity analyses; focus on uncertainty in rates of TB reactivation (using scenario analysis) and TST and IGRA test characteristics (specificity, sensitivity, cost). Other parameters explored: LTBI prevalence, treatment completion, long-term utility decrement for patients with cured TB, screening cost, proportion returning for TST result	X	Test specificity and sensitivity (TST vs IGRA) ^a^	X	X
Kowada 2014 ^26^	One-way SA found CE was sensitive to the sensitivity and specificity of various screening tests for the different screening scenarios. One-way SA used the following parameters: LTBI prevalence of PLWH during pregnancy; TB incidence of PLWH during pregnancy; LTBI prevalence of PLWH during postpartum period; TB incidence of PLWH during postpartum period; TB risk ^b^ during pregnancy; TB risk ^b^ in postpartum period; increased mortality due to active TB in pregnant PLWH; reactivation rate among pregnant PLWH by age group; prevalence of MDR-TB rate; mortality rate by MDR-TB; age-specific all- cause mortality for pregnant PLWH; probability of successful active TB treatment; probability of recurrence of active TB after treatment; effectiveness of PT; PT adherence for IGRA and TST; probability of DILI; sensitivity, specificity and costs of LTBI screening tests; all utilities	X	X	CEAC based on varying the parameters varied in one-way SA (graph not shown)	X
Azadi *et al.* 2014 ^24^	Clinical training costs, utility for TB-HIV coinfection, hazard ratio of TB death associated with the trial intervention (TST screening and PT)	✓	X	Clinical training costs, utility for TB-HIV coinfection, hazard ratio of TB death associated with the trial intervention (TST screening and PT)	✓
Capocci *et al.* 2015 ^25^	Varied: costs of tests, costs of PT and active TB tx, QALY reductions for LTBI and active TB, IGRA sensitivity, screening and PT uptake, PT effectiveness, indeterminate IGRA rate	X ^c^	X	CEACs based on varying: costs of tests, costs of PT and active TB tx, QALY reductions for LTBI and active TB, IGRA sensitivity, screening and PT uptake, PT effectiveness, indeterminate IGRA rate, proportion of black Africans IGRA+, proportion of subjects from middle TB incidence countries IGRA+, proportion of subjects from low TB incidence countries IGRA+	✓
Tasillo *et al.* 2017 ^29^	Parameters varied included LTBI prevalence, test characteristics, ^d^ age of cohort (which relates to remaining life expectancy and cumulative TB risk), quality-of life estimates.	X	X	CEACs based on varying: reactivation rate, LTBI prevalence, test characteristics ^d^	X
Wong *et al.* 2019 ^30^	Scenario analysis: varied levels of ART coverage and LTBI testing and treatment uptake.	X	Screening and PT coverage	CEACs based on varying: LTBI prevalence, PT effectiveness, LTBI test cost, PT cost, ART coverage, LTBI testing and PT coverage	X ^e^
Jo *et al.* 2021 ^31^	One-way SA to describe the association between each input variable and ICER Results only presented for some high-risk groups and only for five input variables with biggest impact on ICER.	✓	X	CEACs and cost-effectiveness plane PSA plots based on varying a large number of parameters over specified distributions	✓ ^f^
Capocci *et al.* 2020 ^32^	One-way SA with halved and doubled cost for TST, T-Spot.TB, CXR, sputum induction, latent, asymptomatic, smear negative, culture positive and active TB treatment, transmission intensity, test uptake and quality of life parameters.	X ^c^	X	CEACs and uncertainty bounds for point estimates generated using parameters drawn from specified probability distributions	✓

CEAC – cost effectiveness acceptability curve; DILI – drug-induced liver injury; MDR – multidrug resistance; PLWH – people living with HIV; PSA – probabilistic sensitivity analysis; PT – LTBI preventive treatment; QALY – quality-adjusted life year; SA – sensitivity analysis; tx – treatment; + – positive. ^a^ Linas *et al.* also varied IGRA test cost for various scenarios of TST test specificity ^27^. ^b^ Difference between “TB risk” and “TB incidence” not stated. ^c^ Systematic one-way sensitivity analysis results presented as a table. ^d^ “Test characteristics” not defined but includes at a minimum test sensitivity, specificity and proportion returning for TST result. ^e^ 95% uncertainty interval presented for sensitivity analysis but not main results. ^f^ Uncertainty bounds presented in manuscript text for Florida (US$282, range: cost-saving to US$11,000) and New York (US$11,265, range: cost-saving to US$119,000) only.

### Quality assessment

Study reporting completeness varied considerably between studies (range 46–88% on CHEERS 25-point checklist, Table 5) with only three studies scoring >80% ^27, 29, 31^ and two studies scoring <60% ^26, 30^. Particularly low-scoring items involved failure to justify model assumptions such as reasons for choice of time horizon, explanations of effectiveness and utility values used and full outlines of estimations of resources and costs. While only three points on the checklist are allotted to explanation of the model used, structural assumptions and analytical methods used (items 15–17), these are crucial to a proper understanding of how each analysis was undertaken, and scores for these items were low (mean 0.39–0.50 across studies). Lacking a complete appreciation of all model assumptions made it difficult to evaluate potential biases in study design. However, of the eight studies conducted in the ART era ^24– 27, 29– 32^, only one included HIV care/ART costs in their analysis, which may push cost-effectiveness estimates up ^29^. Conversely, secondary transmission was included by only five studies ^27– 29, 31, 32^, despite its incorporation driving estimates down.

**Table 5.** Summary of reporting completeness of included studies scored according to the Consolidated Health Economic Evaluation Reporting Standards (CHEERS) checklist ^19^.

Item	Recommendation	Sawert 1998 ^28^	Linas 2011 ^27^	Kowada 2014 ^26^	Azadi 2014 ^24^	Capocci 2015 ^25^	Tasillo 2017 ^29^	Wong 2019 ^30^	Jo 2020 ^31^	Capocci 2020 ^32^	Mean
	***Title and abstract***										
**1**	Title: Identify the study as an economic evaluation or use more specific terms such as “cost-effectiveness analysis”, and describe the interventions compared.	0	0	1	0.5	1	1	0	1	1	0.61
**2**	Abstract: Provide a structured summary of objectives, perspective, setting, methods (including study design and inputs), results (including base case and uncertainty analyses), and conclusions.	0.5	0.5	1	1	0.5	1	1	1	1	0.83
	***Introduction***										
**3a**	Background and objectives: Provide an explicit statement of the broader context for the study.	1	1	1	1	1	1	1	1	1	1.00
**3b**	Background and objectives: Present the study question and its relevance for health policy or practice decisions.	1	1	1	1	1	1	0.5	1	1	0.94
	***Methods***										
**4**	Target population and subgroups: Describe characteristics of the base case population and subgroups analysed, including why they were chosen.	1	1	1	1	1	1	1	1	1	1.00
**5**	Setting and location: State relevant aspects of the system(s) in which the decision(s) need(s) to be made.	1	1	0	1	1	1	1	1	1	0.86
**6**	Study perspective: Describe the perspective of the study and relate this to the costs being evaluated.	0.5	1	0.5	0.5	0.5	0.5	0	1	0.5	0.56
**7**	Comparators: Describe the interventions or strategies being compared and state why they were chosen.	1	1	0	1	1	1	0	1	0.5	0.72
**8**	State the time horizon(s) over which costs and consequences are being evaluated and say why appropriate ^a^	0.5	0.5	0.5	0.5	0.5	0.5	0	1	0.5	0.50
**9**	Report the choice of discount rate(s) used for costs and outcomes and say why appropriate.	1	1	0.5	0.5	1	1	0.5	1	1	0.83
**10**	Describe what health outcomes were used as the measure(s) of benefit in the evaluation and their relevance for the type of analysis performed.	1	1	1	1	1	1	1	1	0.5	0.94
**11**	Measurement of effectiveness: *Single study-based estimates:* Describe fully the design features of the single effectiveness study and why the single study was a sufficient source of clinical effectiveness data. *Synthesis-based estimates:* Describe fully the methods used for identification of included studies and synthesis of clinical effectiveness data.	1	0	0	1	1	0.5	0	0	1	0.50
**12**	Measurement and valuation of preference-based outcomes: If applicable, describe the population and methods used to elicit preferences for outcomes.	0	1	0	1	1	1	0	0	0	0.44
**13**	Estimating resources and costs: Describe approaches and data sources used to estimate resource use associated with model health states. Describe primary or secondary research methods for valuing each resource item in terms of its unit cost. Describe any adjustments made to approximate to opportunity costs.	1	1	0	0.5	0	0.5	0	0.5	0.5	0.44
**14**	Currency, price date, and conversion: Report the dates of the estimated resource quantities and unit costs. Describe methods for adjusting estimated unit costs to the year of reported costs if necessary. Describe methods for converting costs into a common currency base and the exchange rate.	1	1	1	1	1	1	0.5	1	1	0.94
**15**	Choice of model: Describe and give reasons for the specific type of decision-analytical model used. Providing a figure to show model structure is strongly recommended.	1	0.5	0.5	0.5	0	0.5	0.5	1	0	0.50
**16**	Assumptions: Describe all structural or other assumptions underpinning the decision-analytical model.	0	1	0	0	0	1	0.5	1	0	0.39
**17**	Analytical methods: Describe all analytical methods supporting the evaluation. This could include methods for dealing with skewed, missing, or censored data; extrapolation methods; methods for pooling data; approaches to validate or make adjustments (such as half cycle corrections) to a model; and methods for handling population heterogeneity and uncertainty.	0	1	0.5	0.5	0	1	0.5	0	0.5	0.44
	***Results***										
**18**	Study parameters: Report the values, ranges, references, and, if used, probability distributions for all parameters. Report reasons or sources for distributions used to represent uncertainty where appropriate. Providing a table to show the input values is strongly recommended.	0.5	1	0.5	0.5	0.5	1	0	0.5	0.5	0.56
**19**	Incremental costs and outcomes: For each intervention, report mean values for the main categories of estimated costs and outcomes of interest, as well as mean differences between the comparator groups. If applicable, report incremental cost-effectiveness ratios.	0	1	0.5	0	1	1	0.5	0.5	1	0.61
**20**	Characterising uncertainty: *Single study-based economic evaluation:* Describe the effects of sampling uncertainty for the estimated incremental cost and incremental effectiveness parameters, together with the impact of methodological assumptions (such as discount rate, study perspective). *Model-based economic evaluation:* Describe the effects on the results of uncertainty for all input parameters, and uncertainty related to the structure of the model and assumptions.	1	0.5	0	0.5	1	0.5	0	1	0.5	0.56
**21**	Characterising heterogeneity: If applicable, report differences in costs, outcomes, or cost-effectiveness that can be explained by variations between subgroups of patients with different baseline characteristics or other observed variability in effects that are not reducible by more information.	1	1	0	0	1	1	1	1	1	0.78
	***Discussion***										
**22**	Study findings, limitations, generalisability and current knowledge: Summarise key study findings and describe how they support the conclusions reached. Discuss limitations and the generalisability of the findings and how the findings fit with current knowledge.	1	1	0	1	1	1	0.5	1	1	0.83
	***Other***										
**23**	Source of funding: Describe how the study was funded and the role of the funder in the identification, design, conduct and reporting of the analysis. Describe other non-monetary sources of support.	0.5	0.5	1	0.5	0	1	1	1	1	0.72
**24**	Conflicts of interest: Describe any potential for conflict of interest of study contributors in accordance with journal policy. In the absence of a journal policy, we recommend authors comply with International Committee of Medical Journal Editors recommendations.	0	1	1	1	0.5	1	0.5b	1	1	0.78
**Total** **(%)**		**16**. **5** (66%)	**20**. **5** (82%)	**12**. **5** (50%)	**17**. **0** (68%)	**17**. **5** (70%)	**22**. **0** (88%)	**11**. **5** (46%)	**20**. **5** (82%)	**18**. **0** (72%)	**17**. **3** (69%)

For each item, positive responses scored 1 and negative responses scored 0; intermediate scored 0.5. (24-item checklist; total points out of 25.) ^a^ 0.5 score if time horizon is reported but with no justification. ^b^ Conflict of interest stated.

Similarly study quality, as assessed by comparing each included study to the Gates Reference Case for Economic Evaluation ^20^, broadly found the same studies performed well ^25, 27, 31, 32^ and poorly ^26, 30^ as identified by the CHEERS checklist ( Table 6). Generally, studies performed poorly on Gates principles which may only recently have been recognised as important for inclusion in cost-effectiveness analyses, such as discussion of equity considerations and budget impact analysis (which is often performed separately to a cost-effectiveness analysis). Heterogeneity, in terms of exploring differential impacts of interventions within subpopulations, was handled differently by studies depending on the research question. This is because some studies treated PLWH as the primary patient population and evaluated respective subgroups (e.g., CD4 count strata ^28^, migrant status ^26^, country of birth ^25, 32^), while others included PLWH as one of several groups at risk for LTBI (e.g., close contacts of TB patients, migrants, vulnerable populations including homeless, drug users and former prisoners, and individuals with medical comorbidities ^27, 31^. Given the small number of studies included, we could not conduct any formal subanalysis by study quality, but there was no trend in terms of cost-effectiveness by study quality.

**Table 6.** Quality assessment of included studies scored according to the Gates Reference Case for Economic Evaluation ^1^. A full description of the evaluation using the 11 principles, including methodological specifications and reporting standards associated with each principle, are listed in Table E2, Extended Data file. Each study was assigned a percentage score for each principle to reflect how well it complied with all aspects of that principle. Scores were determined independently by RFB and CV and resolved through a consensus meeting.

Principle	Description	Sawert 1998 ^28^	Linas 2011 ^27^	Kowada 2014 ^26^	Azadi 2014 ^24^	Capocci 2015 ^25^	Tasillo 2017 ^29^	Wong 2019 ^30^	Jo 2020 ^31^	Capocci 2020 ^32^	Mean
**1.**	**Transparency.** An economic evaluation should be communicated clearly and transparently to allow the decision-maker(s) to interpret the methods and results **Base Case Analysis:** ● State decision problem using PICO format and describe context of decision ● Outline limitations of analysis in informing health policy Declare interests of study authors and source of funding	50%	90%	30%	30%	30%	100%	20%	70%	30%	50%
**2.**	**Comparators.** The **comparators** against which costs and effects are measured should accurately **reflect the decision problem** **Base Case Analysis:** ● Current practice in context of decision problem to serve as base case comparator ● Do nothing comparator should be explored as additional analysis	70%	100%	10%	10%	100%	100%	20%	20%	100%	59%
**3.**	**Use of Evidence**. An economic evaluation should consider **all available** **evidence relevant to the decision problem.** **Base Case Analysis:** ● Apply systematic and transparent approach to obtaining and using evidence	50%	70%	20%	50%	40%	90%	20%	70%	30%	49%
**4.**	**Measure of outcome.** The **measure of health outcome** should be **appropriate to the decision problem**, should capture **positive and** **negative effects on length of life and quality of life**, and should be **generalisable** across disease states. **Base Case Analysis:** ● Disability Adjusted Life Years (DALYs) averted ***[stated methodological*** ***specification]*** ● Alternative generic (e.g. QALY) health outcome measures encouraged in separate analysis	50%	100%	50%	80%	50%	100%	40%	50%	80%	67%
**5.**	**Measurement of costs.** All **differences** between the intervention and the comparator in **expected resource use and costs** of delivery to the target population(s) should be incorporated into the evaluation. **Base Case Analysis:** ● All relevant direct resource use and costs of implementing intervention to be identified, included donated resources and out of pocket payments (see principle 7)	30%	70%	30%	30%	30%	70%	10%	30%	10%	34%
**6.**	**Time horizon for costs and effects.** The **time horizon** used in an economic evaluation should be of **sufficient length** to capture all costs and effects **relevant to the decision problem**; an appropriate **discount rate** should be used to **discount cost and effects to** **present value.** **Base Case Analysis:** ● Lifetime time horizon (or sufficient to capture all relevant cost and effects) ● Discount rate of 3% for both costs and effects ***[stated*** ***methodological specification]***	25%	90%	90%	25%	90%	90%	0%	80%	90%	64%
**7.**	**Costs and effects outside health. Non-health effects** and **costs associated with gaining or providing access to health** **interventions that don't accrue to the health budget** should be identified where **relevant to the decision problem.** All costs and effects should be **disaggregated,** either by sector of the economy or to whom they accrue. **Base Case Analysis:** ● Reflect direct costs to the health budget and direct health outcomes to patients. ● Include costs incurred by external funders or individual OOP payments where it substitutes for costs that would otherwise accrue to the health budget ● All relevant non– health effects and costs that fall outside health budget to be identified	0%	0%	0%	0%	0%	0%	0%	0%	0%	0%
**8.**	**Heterogeneity.** The **cost and effects of the intervention on sub-** **populations** within the decision problem **should be explored** and the **implications** appropriately **characterised.** **Base Case Analysis:** ● Explore and identify significant population subgroups ● Report separate subgroup analysis where heterogeneity relevant to the decision problem exists	90%	90%	60%	0%	90%	60%	20%	90%	90%	66%
**9.**	**Uncertainty.** The **uncertainty** associated with an economic evaluation should be appropriately **characterised.** **Base Case Analysis** ● Explore all relevant structural, parameter source, and parameter precision uncertainty ● Probabilistic sensitivity analysis preferred but not explicitly required	30%	25%	25%	70%	75%	40%	40%	85%	75%	52%
**10.**	**Impact on other constraints and budget impact.** The **impact** of implementing the intervention on **health budgets and other** **constraints** should be **identified clearly and separately**. **Base Case Analysis** ● Report expected budget impact of implementing the intervention on all relevant budgets in the context for the population identified in the decision problem	0%	0%	0%	0%	0%	0%	0%	0%	0%	0%
**11.**	**Equity implications.** An economic evaluation should explore the **equity implications** of implementing the intervention. **Base Case Analysis** ● Equity implications of implementing the intervention for the populations described in the decision problem should be reported, however the reporting method is at discretion of researcher or the needs of the decision maker	0%	0%	0%	0%	0%	0%	0%	0%	0%	0%

## Discussion

To our knowledge, this is the first systematic review of cost-effectiveness of LTBI screening/PT focussing on PLWH in lower TB incidence settings, and it highlights the limited number of studies published. Cost-effectiveness estimates of LTBI screening/PT for PLWH varied widely: taking studies published in the past five years, which should be relatively similar in terms of assumptions such as ART use, cost-effectiveness of strategies screening all PLWH varied from $2828 to $144,929 (n=5, 2018 prices). Included studies have such variation in strategies evaluated, target populations and methods and assumptions used, that it is hard for policy makers to interpret these results, identifying which model inputs are driving these extreme values and how they relate to their own populations, in order to make informed decisions regarding screening strategies. Strategies targeting screening/PT to PLWH at higher risk of LTBI were found to vary markedly in their cost-effectiveness (NICE 2016 strategy: $131,643/QALY gained, BHIVA 2011: $58,297/QALY gained in the UK ^32^), with alternative strategies found to be more cost-effective ^32^. These findings should be evaluated in conjunction with estimates of number of LTBI cases missed by each strategy in order to devise revised, coherent national guidelines.

Study quality and reporting completeness were assessed using the Gates Reference Case for Economic Evaluation and the CHEERS checklist, respectively. However the insights gained from these were limited because of the heterogeneity between studies. Furthermore, generic measures of study quality may fail to capture which model assumptions are key and are most likely to bias the outcomes, as they are not specifically designed to evaluate or compare epidemiological models. Development of more precise evaluation tools for these types of analyses, where a range of different models may be used to evaluate cost-effectiveness of an infectious disease intervention, will help with model comparison. Such evaluation methods have already been developed for specific model types (infectious disease transmission models) ^47, 48^.

Further research is required to provide the evidence base to inform LTBI screening policies. The many methodological facets listed in Table 1 and Table 2, which are not exhaustive, demonstrate the many factors contributing to study heterogeneity, with several study quality issues also identified, as outlined above. No study considered cost-effectiveness for children living with HIV. LTBI screening for newly HIV-diagnosed should be evaluated separately from catch-up programmes screening those in established HIV care, who are likely to have lower risk of LTBI. The most recent studies by Jo *et al.* ^31^ and Capocci *et al.* 2020 ^32^ come to very different conclusions, and while they are from different settings (US and UK), policy makers from all lower TB incidence settings need to understand the factors driving these differences to develop effective strategies for their own populations. Among these, the high TB mortality rate assumed by Jo *et al.* ^31^ (9.2%) will drive ICER estimates down while the high cost of PT assumed by Capocci *et al.* 2020 ^32^ will drive it up. However, TB mortality assumptions were not recorded by Capocci *et al.* 2020 ^32^, and while Jo *et al.* ^31^ fitted TB prevalence for the screened population to TB incidence data, prevalence estimates are not stated, so the reader cannot compare the two studies on many influential model inputs.

Capocci *et al.* 2015 demonstrated that cost-effectiveness of screening/PT strategies changed markedly over time ^25^. As LTBI prevalence is likely to reduce further with global TB prevention efforts, attention should focus on cost-effectiveness of targeting strategies to populations of PLWH at highest risk of infection and reflect on how cost-effectiveness may change over time as LTBI prevalence hopefully further decreases, as considered by Jo *et al*. ^31^. However, LTBI screening/PT should not necessarily stop as its cost-effectiveness drops, as management of LTBI in high-risk groups including PLWH is a priority for TB control as part of the Global End TB Strategy ^49, 50^. Furthermore, WTP thresholds vary hugely by country ( Figure 2 illustrates the large differences in thresholds assumed by included studies), demonstrating the variation in what is deemed cost-effective, even when restricted to lower TB incidence settings.

While heterogeneity in model structure and assumptions can hamper comparability, it is still important to consider this diversity to explore the full range of uncertainty and identify which aspects, such as incorporating MDR, or onward TB infection transmission, are most influential and therefore important to include. However, a more standardised approach to presentation of methods and results, including systematic and well-justified sensitivity analyses, will facilitate comparisons between studies so that policy makers can fairly judge the evidence available on which to base LTBI screening guidelines in these settings. Items 15–17 of the CHEERS checklist, relating to model structure, assumptions and methods, only contribute three points to the reporting score but we recommend it should be given more weight as they are crucial to understanding how all model inputs relate to the outputs. Lessons can be learned from other fields to develop a descriptive framework to make future cost-effectiveness analyses more rigorous and comparable ^51^.

Assessment of uncertainty is an important aspect of all cost-effectiveness analyses. We found sensitivity analyses conducted by included studies to be highly heterogeneous, and choice of parameters and the ranges through which they were varied were not always rigorously justified, though quality increased over time. To standardise the general reporting of cost-effectiveness analyses, the International Society for Pharmacoeconomics and Outcomes Research (ISPOR) developed the CHEERS reporting checklist [28], which we used to evaluate study quality ( Table 4). We recommend further standardisation of cost-effectiveness analyses to mandate inclusion of Tornado plots with justification of each parameter range used. These provide a more effective, objective summary of the most influential parameters driving model output (as long as parameter ranges are well justified) than lengthy descriptions of results in the text, and all parameters should be included rather than just a selection, subjectively chosen (it is also important to identify which parameters have little impact on study outcomes as these should then have less weight in decision making). While PSA was widely used by included studies to create cost-effectiveness acceptability curves, we would endorse its use to generate uncertainty ranges for ICER estimates.

Improving clarity will further improve the accessibility of studies. We found a lack of precision in description of model parameters sometimes limited our understanding of how they related to model structure and in turn, model output. For example, authors should be clear whether “TB” refers to TB disease (often referred to as active TB) or latent TB infection, and should always specify units and clarify proportions versus percentages. They should state to which population group or subgroup the specific parameters apply, and for each subgroup created (e.g., patients developing DILI, those with MDR) it should be articulated: 1) what proportion of the cohort is in the subgroup, 2) over what duration they remain in this group and 3) how that affects their costs and health benefits. It should be clear, also, how inputs such as treatment adherence affect therapeutic effectiveness, and therefore influence model outputs.

A contentious issue regarding HIV-associated TB is the downstream costs of HIV care. ART is lifelong; therefore, interventions improving survival for PLWH may appear less cost-effective than for HIV-uninfected individuals. Therefore, it is perhaps unsurprising that only one included study accounted for HIV care costs ^29^. Currently, PT for PLWH in low TB incidence (generally higher resource settings) has only a marginal gain in terms of life expectancy (PT nonetheless playing an important role in TB control by reducing morbidity, costs of TB disease treatment and onward TB infection transmission). Therefore, the inclusion or exclusion of ART costs should not be as influential as seen in other contexts ^52– 54^. Nonetheless, it raises important ethical questions regarding the design and interpretation of cost-effectiveness analyses involving increasing the life expectancy of PLWH ^52^.

There are limitations to our analysis. Principally, we could not explore factors driving model output in more detail because of the limited number of studies included. While broadening our focus to include higher TB incidence countries would increase these numbers, the very different contexts (TB reinfection rates, mortality rates, ART coverage and costs, among others) means comparisons between studies would be equally challenging. We are also unable to rule out the possibility of publication bias, with potential selective publication of more favourable cost-effectiveness estimates. Only one of the included studies reported a conflict of interest of the authors (receiving personal fees from pharmaceutical manufacturers ^30^), and selection/omission of model assumptions which would make outcomes more/less favourable (ART costs, secondary transmission) was not uniform across studies. However, Jo *et al.* selected the four states where more than half of US TB cases occur, so cost-effectiveness of screening is likely to be reduced in states with lower prevalence ^31^. These states are also the richest in the US by Gross Domestic Product ^55^.

Our study highlights the need for further research evaluating the cost-effectiveness of LTBI screening/PT, employing the highest standards of methods and reporting in order to make useful contributions to the field that can be used by policy makers to inform national guidelines. As TB prevalence hopefully continues to fall across the world, we need to consider targeting strategies which will be cost-effective now and in the future, to provide good value for the resources invested and better health for PLWH.

## Data Availability

All data underlying the results are available as part of the article and no additional source data are required. Figshare: Extended data for the manuscript: Health economic analyses of latent tuberculosis infection screening and preventive treatment among people living with HIV in lower tuberculosis incidence settings: a systematic review, https://doi.org/10.6084/m9.figshare.13724635.v1 ^17^. Figshare: PRISMA guidelines for “Health economic analyses of latent tuberculosis infection screening and preventive treatment among people living with HIV in lower tuberculosis incidence settings: a systematic review”. https://doi.org/10.6084/m9.figshare.13724635.v1 ^17^. Data are available under the terms of the Creative Commons Zero "No rights reserved" data waiver (CC0 1.0 Public domain dedication). **Declaration of interests:** MP received grants and personal fees from Gilead Sciences and personal fees from QIAGEN, outside the submitted work. RM also received personal fees from Gilead Sciences, outside the submitted work. ML was senior author of two studies included in the review ^23, 30^ and was a member of the BHIVA 2019 TB/HIV Guideline Group and the NICE 2016 TB Clinical Guideline Group; ML played no role in data extraction or assessment of study quality for this review. CV is a consultant for Oriole Global Health. All other authors have nothing to declare. **Author contributions:** All authors have contributed significantly to this work, providing substantive input into the review plan for the PROSPERO submission, interpreting results, and commenting on manuscript drafts. RFB, MP and TDH conceived the study. RFB and CV undertook study screening. RFB extracted the data which was verified by CAD and CV. RFB drafted the manuscript, with MP and TDH providing ongoing advice and consultation on the analysis plan and manuscript preparation.
