# Supplementary material for: Health economic analyses of latent tuberculosis infection screening and preventive treatment among people living with HIV in lower tuberculosis incidence settings: a systematic review
Source: Wellcome Open Res. 2023 Jan 5;6:51. Originally published 2021 Mar 5. [Version 2] doi: 10.12688/wellcomeopenres.16604.2 (PMC10071141; doi:10.12688/wellcomeopenres.16604.2)
Supplement: Peer review history

# Peer review: Reviewer response for version 2

**DOI:** 10.21956/wellcomeopenres.20730.r55882

**Published:** 2023-04-03

**Authors:** Pete Dodd

**Affiliations:** 1 School of Health and Related Research, The University of Sheffield, Sheffield, UK

This is a well put together systematic review of economic analyses of LTBI screening in PLHIV in lower TB incidence settings. In my view previous peer review has improved this and it should be accepted. Below I offer a few minor and optional suggestions:

1. Last sentence of abstract intro 'Evidence...cost.' Taking this literally, it's not quite true that evidence does this. Suggest either deleting 'Evidence of' or changing to 'Evidence suggests..' whichever is closer to intended meaning.
2. Intro 1st sentence: I suppose the latest thinking would suggest changing wording from 'they are infected' to 'they have immunological evidence of exposure' or similar
3. Intro 'long-lasting...mitigated'. Think meaning not quite clear if taking literally. In these countries the benefit of PT may be longer lasting due to re-exposure being less likely. Suggest improving phrasing.
4. Selection criteria: more often called Inclusion criteria?
5. Could the authors comment briefly somewhere (Discussion?) on the rationale for restricting to strategies that involved screening? I see they excluded 1 study on this basis.
6. Could the authors comment briefly somewhere (Discussion?) on the rationale for restricting to studies that were model-based. It seems none were found however.
7. Exclusion criterion 3. Presumably most strategies screened for TB disease before using PT? Perhaps change wording to 'screening for TB disease alone' if this is the meaning?
8. 'dynamic-type Markov models' I think this may confuse readers who interpret this as meaning they are dynamic (ie transmission models). Suggest deleting dynamic-type.
9. Table 1. Suggest adding whether the model is static or dynamic (or includes transmission or not) in each case under the 'Model Type' column.
10. Table 3. Suggest adding setting underneath author for each row of 1st column. I know this data is elsewhere, but I think having it in this table would aid with interpretation of the costs. (Perhaps also 'TB disease' rather than 'Active TB' in the column header)
11. Re Discussion pg 24; suggestion for Results/Discussion. Could the authors briefly comment on how studies typically modelled detection (ie was all TB assumed to be detected & treated)?
12. Re Discussion pg 24; suggestion for Results/Discussion. Could the authors briefly comment on whether studies included costs of contact tracing efforts in relation to future TB?
13. Re Discussion pg 24; suggestion for Results/Discussion. Could the authors briefly comment on typical model/code availability? 

Is the review written in accessible language?

Yes

Are all factual statements correct and adequately supported by citations?

Yes

Are the conclusions drawn appropriate in the context of the current research literature?

Yes

Is the topic of the review discussed comprehensively in the context of the current literature?

Yes

Reviewer Expertise:

TB epidemiology; modelling; model-based economic evaluation

I confirm that I have read this submission and believe that I have an appropriate level of expertise to confirm that it is of an acceptable scientific standard.

# Peer review: Reviewer response for version 2

**DOI:** 10.21956/wellcomeopenres.20730.r53895

**Published:** 2023-02-06

**Authors:** Susmita Chatterjee

**Affiliations:** 1 Department of Research, George Institute for Global Health, New Delhi, Delhi, India

No further comments to make.

Is the review written in accessible language?

Yes

Are all factual statements correct and adequately supported by citations?

Partly

Are the conclusions drawn appropriate in the context of the current research literature?

Partly

Is the topic of the review discussed comprehensively in the context of the current literature?

Partly

Reviewer Expertise:

Health economics

I confirm that I have read this submission and believe that I have an appropriate level of expertise to confirm that it is of an acceptable scientific standard.

# Peer review: Reviewer response for version 1

**DOI:** 10.21956/wellcomeopenres.18301.r43486

**Published:** 2022-04-22

**Authors:** Richard White, Nicola Foster

**Affiliations:** 1 TB Modelling Group, TB Centre, Centre for Mathematical Modelling of Infectious Diseases, Department of Infectious Disease Epidemiology, London School of Hygiene & Tropical Medicine, London, UK

**Brief summary of paper:**

- The authors conducted a systematic review of cost-effectiveness analysis of latent TB infection screening in low TB disease incidence settings (defined as <100 cases per 100 000 people per year). They identified nine studies to include, from the USA, Italy, Japan, Brazil, UK and China.
- They also aimed to assess whether studies were comparable in their conclusions for the cost effectiveness of all PLWH vs targeted screening, and which aspects of the studies most influenced the predictions.

**Summary of our opinion:**

- Very clear and well written paper, and could make an important contribution to current policy decisions in the UK.
- Overall, the evaluation is well structured, with a clear description of study inclusion and exclusion criteria. 
- We would very much like to see the manuscript published after the authors consider making the changes highlighted below

**Data and methods:**

 Methodologically sound. Reported efforts for reducing bias including multiple abstract reviewers.

 Major:

- A definition of <100 cases per 100 000 people per year was used to define low TB incidence settings. The European framework for Low TB incidence countries defines low TB incidence countries as countries with case notification rates of <10 per 100 000 inhabitants and declining. High risk populations are defined as those with a notification rate of >100 cases per 100 000 population (Broekmans et al. 2002. European framework for tuberculosis control and elimination in countries with a low incidence, ERJ, 19:765-775). Brazil has a TB notification rate of 35 cases per 100 000 people per year and China has a notification rate of 43 cases per 100 000 people per year (WHO Global Tuberculosis Report 2021). Therefore, using the definition from the European TB control framework, neither of these countries would be considered low TB incidence countries.
  - Recommend discussing why this definition was used in the paper, referencing other definitions. 
- Authors reviewed the quality of studies included in the review using the CHEERS checklist (Husereau *et al. *2013. Consolidated Health Economic Evaluation Reporting Standard. BMJ, 346: f1049).  Given that the CHEERS checklist is not designed for assessing the quality of a study but rather the completeness of reporting, what the authors are assessing is how well the study was reported and not on the quality of the analytical decisions that were made in conducting the studies and therefore not how ‘credible’ the results are. The Gates reference case or the work of the Gold panel on cost- and cost-effectiveness analyses could have been used to assess the quality of the studies used in the review (Claxton *et al. *2014. The Gates Reference case for economic evaluation. The Bill and Melinda Gates Foundation) and (Weinstein et al. 1996. Recommendations of the panel on cost-effectiveness in health and medicine. JAMA, 276(15):1253-1258). Y
  - Given the amount of work that would be required of the authors to restructure the review to assess the quality of papers included, we recommend updating the explanation by replacing the ‘assessment of study quality with an ‘assessment of the completeness of reporting of studies’, or similar, to the methods, and adding comments on the limitations of the approach to the discussion.
- The review excludes studies reporting costs results only i.e. with no health/ utility component. Assessing the health impacts of LTB screening is challenging and may be under-reporting the utility impact of latent TB (Wong et al. 2021. Impact of latent tuberculosis infection on health and wellbeing: a systematic review and meta-analysis. ERR, 30: 200260). It is therefore unclear in this review why studies not assessing health impact were excluded from the review.
  - Recommend adding a justification in the paper for this exclusion. 
- When comparing results between different studies, the following key issues were noted and our recommendations are outlined:
  - Included studies with different utility outcomes, cost/ DALY averted and cost/QALY gained. 
    - Need to state what efforts were made to make these studies comparable
  - Nor clear if assessed studies differ depending on whether they include a transmission component or not 
    - Need to state the method
  - After extraction of all cost, and cost-effectiveness data, costs were inflation-adjusted to 2018 values, the approach used was however not stated. Populations were from very different health systems and included the US, Italy, Japan, Brazil, UK and China. Does the approach for inflation adjustment take this into account?
    - Need to state the method used by authors to inflate reported costs to a current cost. 
    - Furthermore, need to add to the discussion whether this approach for inflation adjustment took different health systems into account
  - Widely varying time horizons, may be cost-effective over life-time but not 10 years - how was this taken into account?
    - Need to state how this taken into account
- Data analysis section. Suggest replace “so structural model assumptions which may particularly affect outputs, and therefore introduce bias, were evaluated”, with “so structural model assumptions which may particularly affect outputs, and therefore introduce bias, were proposed”, as the evaluation was (as far as we could tell) subjective.

**Interpretation of findings:**

 Major:

 Including ART costs or not including ART costs, was well discussed as an important driver of cost-effectiveness results. The benefits of ART will significantly alter the utility results of the evaluation (decreasing the effectiveness of the IPT) therefore, in our opinion, the costs should also be included. This would increase ICERs though i.e. IPT will be less cost-effective compared to other evaluations.

- In the results of the authors' systematic review, the authors don’t explicitly discuss the quality of the studies even though this was mentioned as an objective earlier in the paper. 
  - Need to add discussion of how the quality of the different studies were assessed and if found to be of low/ high quality, and if/how was this incorporated into the analysis?

The discussion section was well laid out. Important topics were covered such as the inclusion of ART costs. 

 Would also recommend adding discussion of the following:

- Why did you restrict the analysis to only studies with costs and utility data, and how may excluding costing studies have skewed your results or recommendations?
- Based on the current evidence, what is your recommendation to policy makers (specifically in the UK) as stated early in the paper, the purpose of the review is to inform their decision-making?
- Transmission components of the model - unclear how including transmission influenced the results of studies and recommendations for future studies. Is there something to be said about transmission given that the population of interest is PLWH with latent TB in low-TB incident settings where transmission is likely to be low?
- In terms of the guidelines, does it matter what other TB screening policies are in place i.e. for nationals from other countries? For example, is the recommendation for screening PLWHA to change depending on whether all migrants are also screened at the country of origin?
- The systematic review excludes grey literature that may introduce publication bias, skewing the results of this systematic review to studies that are published because they have positive results. Recommend discussing this bias in the limitations section of the manuscript. 

**Clarity:**

 Major:

 Very well written paper, clearly and logically outlines the research questions, analysis and discusses the findings.

- Table 3 lists the key cost components reported in the studies, but it is unclear what the unit of the cost estimate reported was. For example, was the cost of full-course LTBI chemoprophylaxis reported per patient or for all patients assessed in the primary studies? 
  - Need to add the unit to the first row of the cost table in Table 3. If the uncertainty intervals were reported in the studies, it would be good to add those to the table too.
- Figure 2 is a very clear and good representation of the different WTP thresholds for each of the countries. Couple of points for clarification: 
  - Why does Japan have the same WTP as the US?
  - Perhaps these could be group together by country rather?
  - On the x-axis of the figure, could explain when a result will be considered cost-effective or recommended for adoption, i.e. below or above the WTP threshold line?

Minor:

- Table 2 heading.  Suggest replacing ‘TB transmission’ with ‘Mtb transmission’ or ‘TB infection transmission’, as the infection is transmitted, not the disease. This error also occurs in other places in the paper.
- Discussion. Suggest replace “, only contribute three points to the quality score but should be given more weight as they are crucial to understanding how all model inputs relate to the outputs”, with “, only contribute three points to the quality score but we recommend it should be given more weight as they are crucial to understanding how all model inputs relate to the outputs”. 

**Motivation/research question:**

 Major:

 Highly topical research question is currently being considered by policy makers in the UK. 

- Could have been strengthened by also including studies that only reported cost estimates.

- Suggest adding discussion of this limitation fully in the discussion section of the paper.

**Lit review/framing:**

 Major:

 Overall well written and good framing of the research question.

 Minor: 

- Statements regarding the prevalence of latent TB infection should be qualified, and critically evaluated. The size of the truly infected population is a current ‘hot topic’ in TB
  - Add references for the statement that “A quarter of the world’s population has latent TB infection (LTBI), meaning they are infected but do not (yet) have symptoms of tuberculosis (TB) and cannot transmit infection.”

Is the review written in accessible language?

Yes

Are all factual statements correct and adequately supported by citations?

Yes

Are the conclusions drawn appropriate in the context of the current research literature?

Yes

Is the topic of the review discussed comprehensively in the context of the current literature?

Yes

Reviewer Expertise:

NF: economics, especially Tuberculosis; RW: epidemiology, especially Tuberculosis

We confirm that we have read this submission and believe that we have an appropriate level of expertise to confirm that it is of an acceptable scientific standard, however we have significant reservations, as outlined above.

# Peer review: Reviewer response for version 1

**DOI:** 10.21956/wellcomeopenres.18301.r48979

**Published:** 2022-04-13

**Authors:** Susmita Chatterjee

**Affiliations:** 1 Department of Research, George Institute for Global Health, New Delhi, Delhi, India

The authors conducted a systematic review on the economic analysis of latent TB screening and preventive interventions among PLWH in a low TB incidence setting. This is an important topic as there is limited health economic research on latent TB. The paper is nicely written. However, I have a few comments / thoughts on this work.

 My main question to the authors is whether they think that this piece of work is helpful to improve the methods of conducting similar research in the future rather than its use for any policy decision? I ask this question as this research was conducted in a low TB incidence setting – where the number of TB cases is low – expectedly latent TB will also be low. Further, I am not clear from the paper on the burden of HIV in those countries. If that is also low, it is very likely that the interventions will not be cost-effective unless we keep adding MDR TB and other situations. Though I again believe that the incidence of MDR TB will also be very low in those settings. So, the question remains what is the main contribution of this study?

 In this context, I think it would be better if the authors can provide incidence rate of TB, latent TB, MDR TB and HIV in the study countries if reported by the authors. Those can be added in Table 1. I also suggest that the results of the studies should be deleted from Table 1 as the table is on study characteristics. 

**Couple of minor points to clarify**

1. Page 3, last line of 3 ^rd^ paragraph, A uniform, evidence-based national guideline for the UK is required. Is this authors’ conclusion? Or a conclusion from other studies? If it's the authors’ conclusion, it should not be here – if other study conclusions, reference should be added.
2. Please check the definition of low TB incidence – as per the current Global TB Report, it is less than 10 cases per 100,000 population per year.

Is the review written in accessible language?

Yes

Are all factual statements correct and adequately supported by citations?

Partly

Are the conclusions drawn appropriate in the context of the current research literature?

Partly

Is the topic of the review discussed comprehensively in the context of the current literature?

Partly

Reviewer Expertise:

Health economics

I confirm that I have read this submission and believe that I have an appropriate level of expertise to confirm that it is of an acceptable scientific standard, however I have significant reservations, as outlined above.
